# Effect of Transition Metal Ions on the B Ring Oxidation of Sterols and their Kinetics in Oil-in-Water Emulsions

**DOI:** 10.1038/srep27240

**Published:** 2016-06-22

**Authors:** Baiyi Lu, Yinzhou Hu, Weisu Huang, Mengmeng Wang, Yuan Jiang, Tiantian Lou

**Affiliations:** 1 College of Biosystems Engineering and Food Science, Fuli Institute of Food Science, Zhejiang Key Laboratory for Agro-Food Processing, Zhejiang R&D Center for Food Technology and Equipment, Key Laboratory for Agro-Food Risk Assessment of Minstry of Agriculture, Zhejiang University, Hangzhou, 310058, China; 2 Zhejiang Economic & Trade Polytechnic, Department of Applied Technology, Hangzhou 310018, China

## Abstract

This study investigated the effect of metal ions on the oxidation of sterols and their kinetics in oil-in-water emulsions. Sterol substrates were added with different metal ions (Cu^2+^, Fe^2+^, Mn^2+^, Zn^2+^, Na^+^, and Mg^2+^) of five concentrations and investigated after 2 h of heating at 90 °C. The substrates added with Fe^2+^ and Cu^2+^ were heated continuously to evaluate the kinetics of four sterols and their corresponding sterol oxidation products (SOPs). Sterol oxidation increased as the metal ion concentration increased and the heating time was prolonged. The capability of the metal ions oxidizing sterols ranked as followed: Fe^2+^ > Cu^2+^ > Mn^2+^ > Zn^2+^ > Mg^2+^ ≈ Na^+^. 7-Ketosterol, 7β/7α-Hydroxysterol, 5β,6β/5α,6α-Epoxysterol, and Triols were the main oxides on the B ring, whereas 6β-Hydroxysterol was not or only slightly influenced. The acceleration of sterol degradation induced by Fe^2+^ and Cu^2+^, as well as the formation of oxidation products, followed first-order formation/elimination kinetics. The acceleration effect may be partly ascribed to the increase in elimination rate constant and formation rate constant. Transition metal ions can significantly induce sterol oxidation, which reduces food nutritional quality and triggers the formation of undesirable compounds, such as SOPs.

Sterols, such as cholesterol and phytosterols, consist of alicyclic alcohols with an unsaturated bond between C5 and C6, a hydroxyl group at C3, and a side chain at C17^1^. Sterols are important functional components in fats and oils, but the inherent molecular structures of these components render them vulnerable to oxidation and subsequent transformation into sterol oxidation products (SOPs)^2^,^3^. The most common SOPs in food are mainly oxidized on the B ring of the sterols, such as 7α/7β-Hydroxysterols, 5α,6α/5β,6β-Epoxysterols, 7-Ketosterol, and triols^4^. Sterol oxidation not only reduces the nutritional quality of food^5^ but also promotes the formation of compounds that exhibit pathological and toxic effects compared with unoxidized sterols. Compared with products of unoxidized sterol, cholesterol oxidation products (COPs) display stronger cytotoxic^6^, apoptotic^7^, and pro-inflammatory effects^8^ and induce atherosclerosis^9^. SOPs are structurally similar to COPs and thus have similar potential hazards to the human body.

Oil-in-water emulsion is a common form of lipids in food, such as butter, margarine, milk, infant formula, salad dressing, mayonnaise, sauces, soups, beverages, cream, and some desserts^10^. These emulsions accelerate lipid oxidation because their large surface areas facilitate interactions between lipids and water-soluble pro-oxidants^11^. The rate of lipid oxidation in oil-in-water emulsions is influenced by numerous factors, the most important of which is metal ions^12^,^13^. Lipid oxidation reduces the nutritional quality of food and triggers the formation of undesirable compounds, such as formaldehyde and acrolein^14^. Metal ions, particularly transition metal ions, form complexes and catalyze lipid oxidation through the homolytic cleavage of hydrogen peroxides and/or direct attack on lipids; in the process, fatty acid radicals are generated to stabilize intermediary species, such as those possibly formed during the thermal oxidation of oils^14^,^15^,^16^. The effects of metal ions are as follows:

1. Accelerating the decomposition of hydrogen peroxide


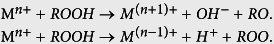


2. Directly oxidizing with material


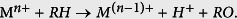


3. Accelerating molecular oxygen into singlet oxygen and oxygen free radicals


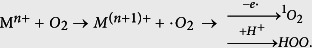


Much effect has been exerted to understand how metal ions accelerate the oxidation of unsaturated fatty acids in oil-in-water emulsions^11^,^17^. However, few studies have focused on the factors that influence sterol oxidation in emulsified oils.

Kinetic studies describe the formation and elimination of chemical reactions. By comparing model types and reaction constants, we can draw a kinetic curve that elucidates and speculates the changes in growth and decline processes. Khuwijitjaru, P. *et al*. found that the loss of γ-oryzanol and its four components (cycloartenyl ferulate, 24-methylene cycloartanyl ferulate, campesteryl ferulate, and b-sitosteryl ferulate) could be described by a first-order kinetics model^18^. Similarly, Castaneda, E. J. L. *et al*.^19^ and Igoumenidis, P. E. *et al*.^20^ found that phytosterol degradation also followed a first-order kinetics model. However, only few kinetic behavior studies focused on the formation and elimination of SOPs. Barriuso, B. *et al*.^21^ reported the degradation and formation of the ring structures of polar oxidation products after heating sterols, where three models were chosen to describe the degradation and formation curve of the corresponding sterols and oxides. However, their study lacked in-depth discussions on reaction rate and mechanism. Fortunately, many dynamic models have been used in similar compounds. These models, which were mainly derived from dynamic mass balance considerations or network reaction pathways and generally fitted based on the hypothesis that all of the intermediate reactions follow first-order kinetics and consequently have characteristic rate constants^22^, could be used as a reference in the study of the formation and elimination of sterol and SOPs.

This work aims to investigate the effect of different metal ions on sterol B ring oxidation in oil-in-water emulsions under continuous heating and to analyze the kinetics of sterol and SOP formation and reduction. In addition, gas chromatography–fire ion detector (GC–FID) and gas chromatography–mass spectrometry (GC–MS) were used for the rapid quantification of sterols and SOPs in reaction products.

## Results and Discussion

### Transformation of sterol and SOPs by the addition of different metal ions

**Figure 1:**
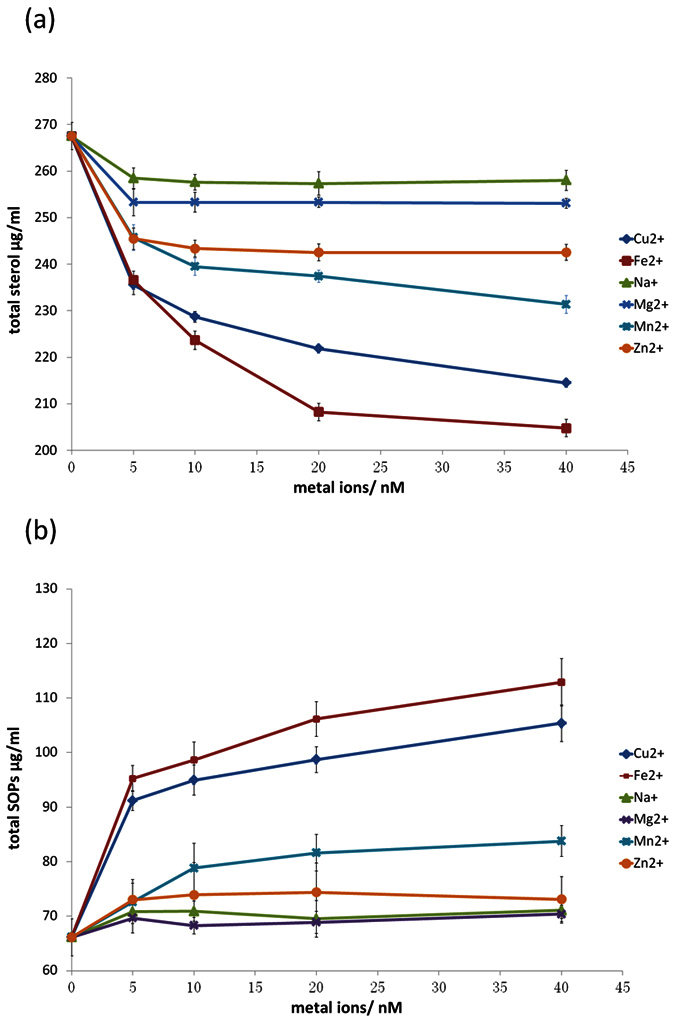
Total sterols and SOPs in the samples with metal ions in different kinds and concentration (**a**) total sterols; (**b**) total SOPs.

The remaining total sterol and SOP contents in samples supplemented with different metal ions at five concentrations after 2 h of heating at 90 °C was summarized in Fig. 1. As shown in Fig. 1a, metal ions were able to accelerate sterol degradation even at a low concentration of 5 nM. The highest amount of sterol loss was found in those samples with the highest concentrations (40 nM) of corresponding metal ions. Such result indicated that total sterol degradation was directly proportional to the concentration of metal ions. This observation was similar to that reported by Bastos, L. C. and P. A. Pereira^14^. In terms of the effect of the different metal ions, Fe^2+^ and Cu^2+^ induced the most sterol losses, followed by Mn^2+^ and Zn^2+^, whereas Mg^2+^ or Na^+^ induced the smallest amount of sterol loss; the remaining sterol contents were 204.78, 214.50, 231.34, 232.51, 253.06, and 258.02 μg/mL of emulsion, at the highest concentration (40 nM) of the corresponding metal ions, respectively.

Heating the emulsion with sterols increased the content of SOPs in all samples (Fig. 1b); moreover, this variation depended on the applied metal ions and their addition level. As the samples were heated, the SOPs increased in the presence of the transition metal ions Fe^2+^, Cu^2+^, and Mn^2+^; the higher the metal ion concentrations were, the greater the observed increase was. However, the formation of total SOPs in the samples with Mg^2+^ or Na^+^ had no significant difference from those in the control samples. The total content of SOPs ranged from 66.12 μg/mL emulsion in the sample without metal ions to 112.88 μg/mL emulsion in the sample with 40 nM Fe^2+^. The SOP contents in the samples with other metal ions were sequenced as Fe^2+^ > Cu^2+^ > Mn^2+^ > Zn^2+^ > Na^+^ ≈ Mg^2+^ at 112.88, 105.37, 83.76, 73.11, 71.06, and 70.37 μg/mL emulsion at the highest concentration (40 nM) of the corresponding metal ions, respectively.

**Figure 2:**
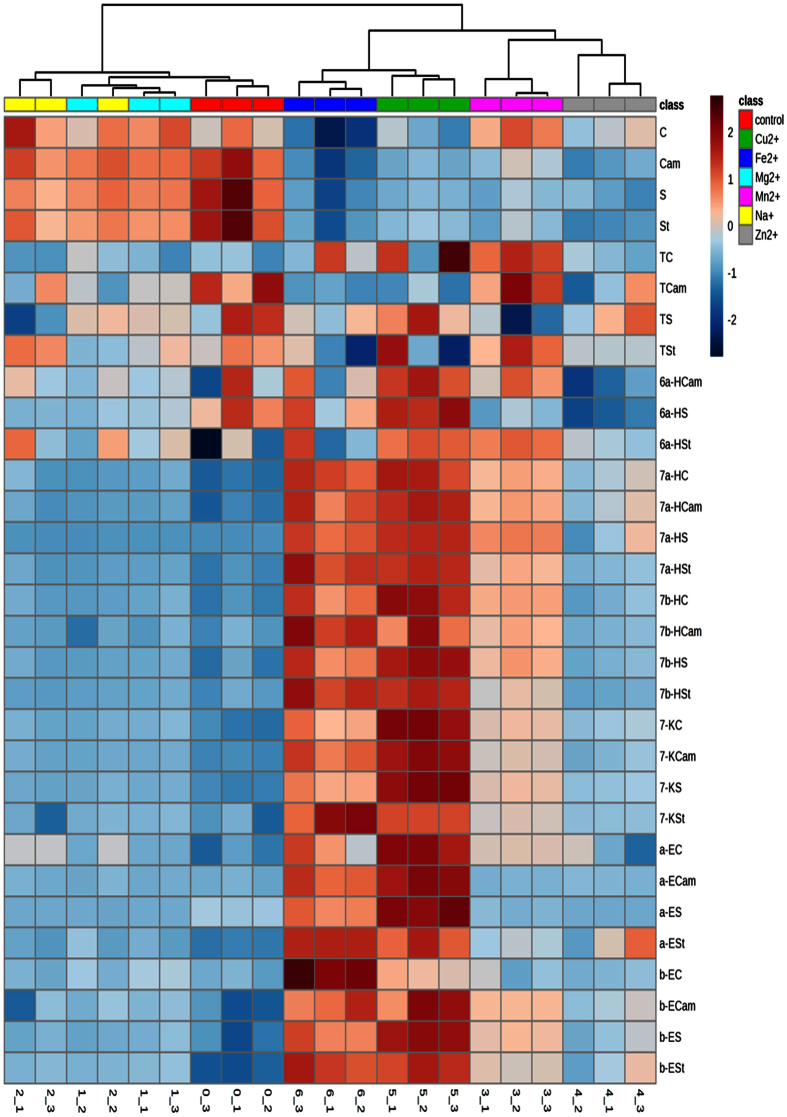
Heat map and cluster analysis of each sterol and their oxidation products in the samples with in different kinds of metal ions at the concentration of 40 nM. C, cholesterol; B, brassicasterol; Cam, campesterol; St, stigmasterol; S, sitosterol; C, cholesterol; H stands for OH; K stands for keto; E stands for epoxy; T stands for triol.

To investigate the distributions of the different metal ion groups, a heat map of free sterol and SOP profiles of the samples with different metals at the highest concentration are illustrated in Fig. 2. In the heat map, the similarity measure is the Euclidean distance, whereas the clustering algorithm is Ward’s linkage by clustering to minimize the sum of squares of any two clusters. As shown in the heat map, the variable distributions of the seven groups are as follows. (a) The effect of metal ions on sterol degradation and SOPs can be divided into two groups, namely, transition metal ions and no-transition ones; the transition metal ions could significantly accelerate sterol degradation as well as SOP formation and elimination. (b) 7-Keto derivatives were the most abundant oxides, followed by 7-hydroxides and 5,6-epoxides (data not show), which was similar to previous reports^2^,^23^,^24^; the remaining derivatives, including 6β-Hydroxy and triols, were not significantly affected by the various metal ions at different concentrations. (c) Moreover, β-epoxides were found in higher amounts compared with α-isomers because of the steric hindrance in position 3 25. 7β-Hydroxides were also found in higher amounts compared with 7α-hydroxides, but the mechanism need to be discovered in further research.

### Kinetic study of sterol degradation and SOP formation and elimination by optimized addition of Cu^2+^ and Fe^2+^

**Figure 3:**
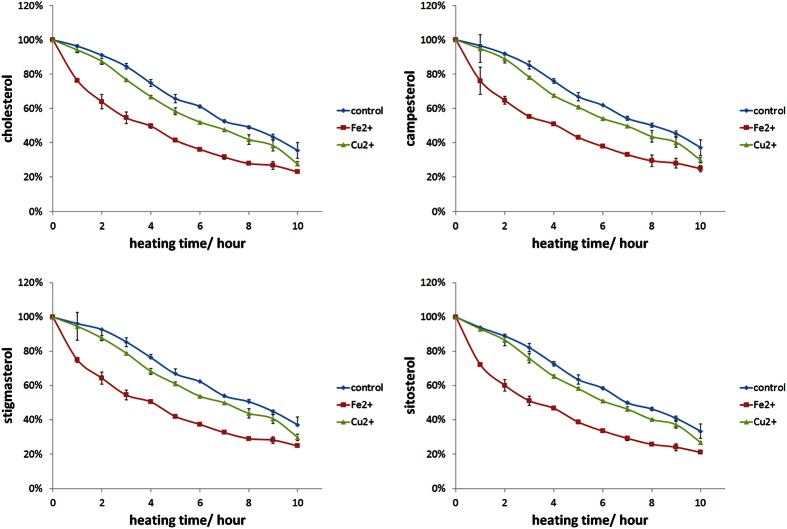
Remaining percentage of sterol in Emulsion sample without metal ions, with 40 nM Fe^2+^ or Cu^2+^, during heating at 90 °C for up to 10 h. (**a**) cholesterol, (**b**) campesterol, (**c**) stigmasterol and (**d**) sitosterol.

**Figure 4:**
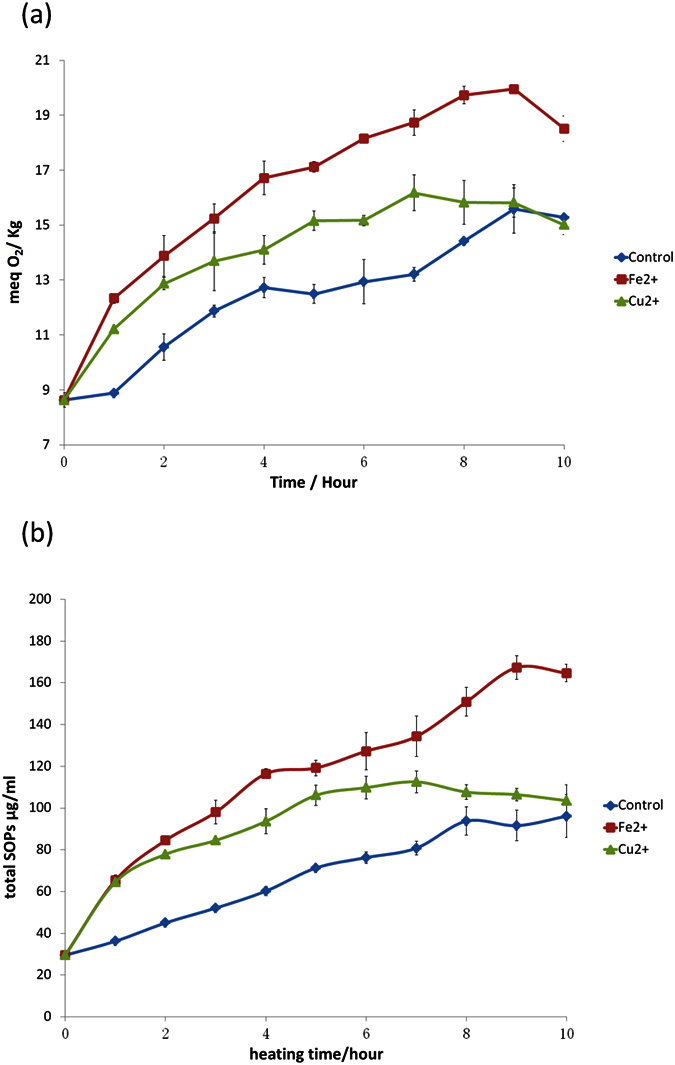
Peroxides Value and total plant sterol oxidation products in Emulsion sample without metal ions, with 40 nM Fe^2+^ or Cu^2+^, during heating at 90 °C for up to 10 h. (**a**) Peroxides Value (meq O2/kg); (**b**) total plant sterol oxidation products.

Figure 3 showed the remaining percentage of each sterol as a function of heating time with and without Cu^2+^ or Fe^2+^. The sterol loss increased as heating time was prolonged in all three treatments. The maximum loss was observed in the samples supplemented with 40 nM Fe^2+^, followed by the samples with 40 nM Cu^2+^. Meanwhile, the highest remaining sterol content was found in control samples. As expected, a similar discipline was found in the changes of Peroxide value (PV) and SOPs (Fig. 4). SOP content increased from 29.54 μg/mL at the beginning to the maximum value 96.17 μg/mL in the control samples after 10 h of heating. The peak value (121.80 μg/mL) was obtained after heating the sample with Cu^2+^ for 6 h, and 167.32 μg/mL was achieved after heating the sample with Fe^2+^ for 9 h. The amount of SOP decreased as the heating time was prolonged, which was in accord with the findings of Barriuso, B. *et al*.^21^. This phenomenon may be attributed to the higher rate of SOP decomposition than SOP formation, which may generate sterene derivatives as Derewiaka, D. *et al*. reported^26^. We also found that PV and SOP content changed simultaneously, which was consistent with the reports of Barriuso, B. *et al*.^24^.

Sterol degradation through application of various methods could be partly ascribed to changes in kinetic behavior. Several mathematical models with different degrees of complexity have been applied to draw a kinetic curve elucidating the reduction of sterols. The optimal model and kinetic parameters for the remaining sterols are shown in Table 1. The degradation of the four sterols clearly fitted a first-order elimination kinetics curve in the three types of samples, with R^2^ values over 0.97 in all cases. Similar results could be found in the studies of Barriuso, B. *et al*.^21^, Igoumenidis, P. E. *et al*.^20^, and Castaneda, E. J. L. *et al*.^19^. However, the R^2^ values in our study were much higher than those from previous works. This result indicates that the model we selected may be the most suitable. Comparisons among the different degradation percentages of sterols showed their increasing susceptibility to oxidation in the samples with metal ions than in the control samples. The degradation speed was calculated as elimination rate constant (k_E_), which increased along with the control, Cu^2+^, and Fe^2+^. The k_E_ of the sample added with Fe^2+^ was 33.3–34.8% higher than that of the control group, whereas that of the sample added with Cu^2+^ was 14.6–18.4% higher than that of the control group.

**Figure 5:**
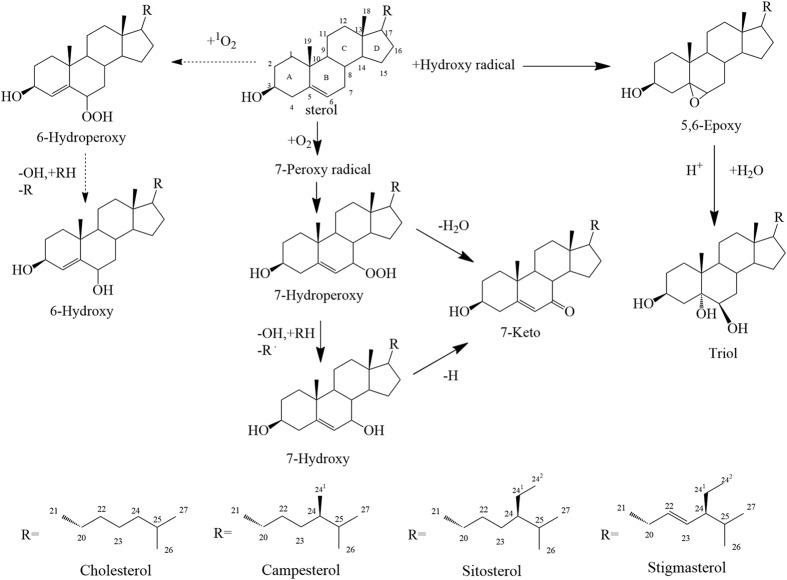
Acceleration of transition metal ions on the B ring oxidation of sterol.

As previously mentioned, the variation of SOP involves two processes, namely, formation and elimination; these processes could be partly ascribed to the change in kinetic behavior. Three mathematical models with different degrees of complexity were used to draw a kinetic curve elucidating the growth and decline of the SOPs. The optimal model and kinetic parameters for SOPs are shown in Table 1. Results indicated that the SOPs (except for 6-Hydroxy) may fit the first-order formation kinetics, with R^2^ values over 0.850 in all cases (with few exceptions). The accelerated degradation and formation of SOPs induced by Fe^2+^ and Cu^2+^ may be partly ascribed to the increase in the formation rate constant (k_F_) of their oxidation products, which increased along with the control, Cu^2+^, and Fe^2+^. Generally, the k_F_ of SOPs in the sample added with Fe^2+^ was higher than the sample added with Cu^2+^. However, the primary oxidation products such as 7-Hydroxy and 5,6-Epoxides may be further oxidized to 7-Keto and triols (as shown in Fig. 5). The acceleration of further oxidation products formation must lead to elimination of the primary oxidation products. This is why the k_F_ of 7-Hydroxy and 5,6-Epoxides in sample added with Fe^2+^ were less than sample added with Cu^2+^.

Sterol oxidation is a free radical mechanism^10^ that can be initiated by several factors, among which transition metals may be the most important^27^,^28^. According to Reaction 1 shown in Section of Introduction, transition metals can react with hydrogen peroxides and yield alkoxide and peroxyl radicals that may propagate the oxidation processes^29^ (Fig. 5). As introduced in Reaction 2, transition metals may induce reactive allylic hydrogen at C7, which is easily autoxidized by triplet oxygen (^3^O_2_) and gives rise to a series of A and B ring oxidation products. The peroxyl radical may react with other lipids to promote peroxidation and the reactions with other radicals in oil, which produces highly reactive and toxic secondary species, such as aldehydes and Ketones^30^. The formation of epoxides by autoxidation occurs through a bimolecular mechanism that includes one hydroperoxy radical and one unoxidized sterol molecule; the formation of both α-epoxides and β-epoxides has been reported. Therefore, we deduce that the acceleration of sterol oxidation induced by transition metal ions was mainly caused by the formation of oxygen free radicals and by the attack on the functional site at C7 and at C=C between C5 and C6. However, the open loop effect of the metal ions on the epoxy compounds, as well as the attack of free radicals on C6, was not evident.

In conclusion, sterol oxidation increased as the metal ion concentration was increased and the heating time was prolonged. In addition, the ability of metal ions to oxidize sterol followed the decreasing order: Fe^2+^ > Cu^2+^ > Mn^2+^ > Zn^2+^ > Mg^2+^ ≈ Na^+^. 7-Ketosterol, 7β/7α-Hydroxysterol, 5β, 6β/5α, 6α-Epoxysterol, and triols were the main oxides on B ring induced by metal ions during heating, whereas 6β-Hydroxysterol was not or only slightly influenced. The acceleration of sterol degradation induced by Fe^2+^ and Cu^2+^, as well as the formation of oxidation products, followed the first-order formation/elimination kinetics. This result may be partly ascribed to the increase in k_E_ and k_F_. We can conclude from the experimental results that transition metal ions can significantly induce free radicals and accelerate sterol autoxidation on C7 and on C=C between C5 and C6 at low concentrations; however, the open loop effects on the epoxy bond and the attack of free radicals on C6 were not influenced. Transition metal ions are important factors in sterol degradation, especially in emulsions during cooking, because they can reduce the nutritional quality of food and lead to the formation of undesirable compounds, such as SOPs.

## Materials and Methods

### Standards and reagents

Olein, stigmasterol, β-sitosterol, campesterol, brassicasterol, cholesterol, 7α-Hydroxycholesterol, 7β-Hydroxycholesterol, 7-Ketocholesterol, 5α,6α-Epoxy-cholesterol, 5β,6β-Epoxycholesterol, cholestanetriol, 19-Hydroxycholesterol, and 5α-cholestane were purchased from Sigma–Aldrich. The derivatization reagents N-methyl-N-(trimethylsilyl) heptafluorobutyramide and 1-methyl imidazole were ordered from Sigma–Aldrich. Acetone, diethyl ether, dichloromethane, hexane, and methanol were obtained from Merck & Co, Inc. FeSO4·7H_2_O, CuSO_4_·5H_2_O, ZnSO_4_, MgSO_4_, Na_2_SO_4_, and MnSO_4_ were analytically pure.

### Sample preparation

Oil-in-water emulsions were prepared by mixing olein (5.0 g/100 mL), sterol (phytosterols or cholesterol 40 mg/100 mL), and Tween 20 (0.6 g/100 mL) with water by using a French press homogenizer (Emulsiflex-C5; Avestin Inc., Canada) at a pressure of 500 bars. During each step of the emulsion preparation, the samples were covered as tightly as possible to avoid exposure to light and were kept in an ice bath. The sizes of emulsion droplets were measured by dynamic light scattering (Zetasizer Nano-ZS, model ZEN90, Malvern Instruments, Worchester, U.K.) and were expressed as the Z-average mean diameter at 1 ± 0.55 nm. No significant change was observed in the droplet size of each emulsion over the whole course of the study (data not shown).

Then, 400 μL of the emulsions were added to 15 mL tubes with stoppers. The emulsions were supplemented with 100 μL of metal ion solutions, including FeSO_4_, CuSO_4_, ZnSO_4_, MgSO_4_, Na_2_SO_4_, and MnSO_4_ at 0.025, 0.05, 0.10, and 0.2 mM. The control group was added with equal volumes of water, and the samples were vortex blended and heated at 90 °C for 2 h. The optimized levels of Fe^2+^ and Cu^2+^ that can exert the maximum reduction rate of sterol among the prepared concentration sequence were selected for the kinetic study. Then, 100 μL of 40 nM Fe^2+^/Cu^2+^, which was the optimized value, was added into the oil-in-water emulsions of the test groups. Most of the procedures were similar to those of the study mentioned above. The mixing solutions in sealed tubes were heated under the selected heating temperature (90 °C after the same programming with different heating durations (0, 1, 2, 3, 4, 5, 6, 7, 8, 9, and 10 h) in different test groups. At each time, the treatment was performed in triplicates (n = 3). The heated samples were quickly cooled down to room temperature and stored at −20 °C without direct light.

### Peroxide analysis

Peroxide value (PV) was determined in accordance with an optimized method described by Shantha and Decker^31^. Emulsion samples (5 μL) were added to a 96-well plate and mixed with 295 μL of a methanol/1-butanol solution (2:1, v/v), resulting in a final volume of 300 μL. The solution was mixed 10 times through suction and blowing. A thiocyanate/ferrous solution was prepared by mixing 500 μL of 3.94 M thiocyanate solution with 500 μL of 0.072 M Fe^2+^ solution, which was obtained from the supernatant of a mixture comprising 1.5 mL of 0.144 M FeSO_4_ and 1.5 mL of 0.132 M BaCl_2_ in 0.4 M HCl. The thiocyanate/ferrous solution was diluted to one over fifteen by methanol/1-butanol (2:1, v/v). Afterward, 170 μL of the thiocyanate/ferrous solution was added to 30 μL of sample diluent, mixed 10 times through suction and blowing, and then incubated at room temperature for 20 min. Following the incubation period, the optical densities of the samples were read at 510 nm on a Spectra Max Plus 384 microplate reader (Molecular Devices, Switzerland). Hydroperoxide content was determined using a standard curve prepared with known concentrations of cumene hydroperoxide. Concentrations were expressed as meq/kg of oil.

### Analysis of sterols

Sterols were analyzed using the procedure optimized from what reported by Rudzin’ ska *et al*.^23^. In brief, emulsion samples (200 mg) with the internal standard 5a-cholestane (50 μg) were saponified with 1 M KOH in ethanol at room temperature for 18 h. The unsaponifiable fraction was extracted with diethyl ether, and the solvent was evaporated under nitrogen. Sterols were silylated in 100 μL of MTBSTFA with 5% 1-MIN for 20 min at 75 °C and then analyzed in an Agilent 7890 GC (Agilent Technologies, USA) equipped with a DB-5 (30 m × 0.25 mm × 0.25 μm, Agilent Technologies, USA) capillary column. Analysis parameters were as follows. The oven temperature was initially set at 180 °C for 1 min, gradually raised to 290 °C at a heating rate of 40 °C/min, and held at 290 °C for 10 min. The injector temperature was 290 °C and the detector (FID) temperature was 310 °C. The carrier gas nitrogen was used at a flow rate of 1.6 mL/min. The sterols were identified by comparing their retention times (relative to 5α-cholestane) with those of commercially available standards.

### Analysis of SOPs

SOP analysis was performed in accordance with an optimized method developed in our laboratory^32^. In brief, emulsion samples (approximately 200 mg) were dissolved in 3 mL of dichloromethane with the internal standard 19-Hydroxycholesterol (5 μg) and shaken for 18 h in the dark at room temperature. The unsaponifiable fraction was extracted with dichloromethane, purified by a single-step SPE, and finally incubated in 100 μL of MTBSTFA with 5% 1-MIN for 20 min at 75 °C. Individual SOPs were analyzed by a 7890A-5973N GC-MS system (Agilent Technologies, USA) equipped with a DB-5MS (30 m × 0.25 mm × 0.25 μm, Agilent Technologies, USA) supplied with a 2 m guard column. SOP separation was attained under the following GC-MS conditions and monitored in SIM model. Helium carrier gas was used at a flow rate of 1.2 mL/min. The oven temperature was initially set at 100 °C for 1 min; gradually raised to 200 °C at 50 °C/min, 250 °C at 20 °C/min, and 300 °C at 1.5 °C/min; and held for 10 min. Injection was hot splitless at 300 °C. The ion source temperature was set at 250 °C, and the transfer line was at 300 °C.

### Statistical analysis

All analyses were carried out in triplicate, and the mean results were reported. Kinetic studies on the calculation of two key rate constants and the precision of fitting kinetic models were statistically evaluated using the 1stOpt Inst. software, version 15 pro (7D-Soft High Technology Inc., China) in accordance with previous publications with slight modifications^21^,^22^.

## Additional Information

**How to cite this article**: Lu, B. *et al*. Effect of Transition Metal Ions on the B Ring Oxidation of Sterols and their Kinetics in Oil-in-Water Emulsions. *Sci. Rep.* **6**, 27240; doi: 10.1038/srep27240 (2016).

## Supplementary Material

Supplementary Information

## Figures and Tables

**Table 1:** Kinetic parameters for remaining sterol and SOPs in Emulsion sample without metal ions, with 40 nM Fe^2+^ or Cu^2+^, during heating at 90 °C.

SOP or Sterol	Ions	Regression model	a	b	k_F_	k_E_	R^2^
Cholesterol	control	a	–	64.301	–	0.103	0.980
	Cu^2+^	a	–	63.437	–	0.122	0.979
	Fe^2+^	a	–	51.797	–	0.14	0.983
Campesterol	control	a	–	22.758	–	0.099	0.981
	Cu^2+^	a	–	22.495	–	0.116	0.980
	Fe^2+^	a	–	18.307	–	0.133	0.981
Stigmasterol	control	a	–	19.671	–	0.099	0.979
	Cu^2+^	a	–	19.357	–	0.115	0.979
	Fe^2+^	a	–	15.603	–	0.132	0.977
Sitosterol	control	a	–	61.035	–	0.109	0.984
	Cu^2+^	a	–	60.621	–	0.125	0.982
	Fe^2+^	a	–	48.072	–	0.147	0.979
5α,6α-Epoxy	Control	b	47.432	43.263	0.044	–	0.987
	Cu^2+^	b	23.957	19.305	0.357	–	0.928
	Fe^2+^	b	40.768	35.553	0.152	–	0.957
5β,6β-Epoxy	Control	b	27.094	15.328	0.082	–	0.979
	Cu^2+^	b	22.272	9.662	0.443	–	0.915
	Fe^2+^	b	30.005	17.067	0.270	–	0.935
7α-Hydroxy	Control	b	26.231	17.643	0.121	–	0.986
	Cu^2+^	b	24.177	13.116	0.711	–	0.949
	Fe^2+^	b	27.087	15.706	0.530	–	0.964
7β-Hydroxy	Control	b	38.409	29.467	0.107	–	0.988
	Cu^2+^	b	31.183	19.466	0.652	–	0.938
	Fe^2+^	b	36.958	24.529	0.464	–	0.976
7-Keto	Control	c	7.626	–	14.246	–	0.888
	Cu^2+^	c	15.403	–	15.501	–	0.891
	Fe^2+^	c	12.352	–	31.165	–	0.854
Triol	Control	c	9.314	–	0.430	–	0.673
	Cu^2+^	c	9.426	–	1.177	–	0.861
	Fe^2+^	c	9.159	–	1.880	–	0.766

a, 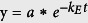; b,
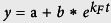
; c, y = a + k_*F*_  * log(*t*).
