# Supplementary material for: Effect of Transition Metal Ions on the B Ring Oxidation of Sterols and their Kinetics in Oil-in-Water Emulsions
Source: Sci Rep. 2016 Jun 22;6:27240. doi: 10.1038/srep27240 (PMC4916447; doi:10.1038/srep27240)
Supplement: Supplementary Information [file srep27240-s1.pdf]

Effect of Transition Metal Ions on the B Ring Oxidation of Sterols and their Kinetics in Oil-in-Water Emulsions

Baiyi Lu[1,*], Yinzhou Hu[1], Weisu Huang[2], Mengmeng Wang[1], Yuan Jiang[1], Tiantian Lou[1]

Table S1 Metal elements in the commercial oil (n=3)

Metal	Rapeseed oil 1 (mg/kg)	Rapeseed oil 2 (mg/kg)	Blend oil 1 (mg/kg)	Blend oil 2 (mg/kg)	Peanut oil 1 (mg/kg)	Peanut oil 2 (mg/kg)	Soybean oil 1 (mg/kg)	Soybean oil 2 (mg/kg)
Fe	0.226±0.001	0.153±0.009	0.152±0.007	0.142±0.014	0.618±0.018	0.173±0.008	0.466±0.017	0.120±0.008
Cu	0.064±0.004	0.042±0.007	0.061±0.013	0.082±0.006	0.127±0.013	0.075±0.012	0.095±0.012	0.041±0.010
Co	ND	ND	ND	ND	ND	ND	ND	ND
Ni	ND	ND	ND	ND	0.003±0.002	0.003±0.001	ND	ND
Pb	ND	0.011±0.003	0.004±0.003	0.045±0.009	0.14±0.001	0.006±0.003	ND	ND
Al	ND	ND	ND	ND	0.222±0.005	ND	ND	ND
Zn	0.037±0.013	0.030±0.004	0.026±0.005	0.064±0.013	0.072±0.004	0.580±0.071	0.062±0.012	0.033±0.007
Cd	ND	ND	ND	ND	ND	ND	ND	ND
Cr	ND	ND	ND	ND	ND	ND	ND	0.002±0.001
Mn	0.052±0.003	0.037±0.003	0.036±0.004	0.032±0.003	0.143±0.008	0.047±0.005	0.038±0.005	0.012±0.002
Mg	0.246±0.002	0.113±0.008	0.059±0.004	0.116±0.009	3.210±0.100	0.067±0.005	0.604±0.014	0.047±0.012

ND, not detected
